# Measuring Processes of Integrated Care for Hospital to Home Transitions

**DOI:** 10.5334/ijic.5552

**Published:** 2021-04-26

**Authors:** Cara L. Brown, Verena Menec

**Affiliations:** 1 Department of Occupational Therapy, Rady Faculty of Health Sciences, University of Manitoba, R106 – 771 McDermot Avenue, University of Manitoba, Winnipeg, MB R3E 0T6, Canada; 2 Department of Community Health Sciences, Max Rady College of Medicine, Rady Faculty of Health Sciences, University of Manitoba, S111-750 Bannatyne Avenue, University of Manitoba, Winnipeg, MB R3E 0W3, Canada

**Keywords:** hospital, discharge planning, older adults

## Abstract

**Background:**

Integrated care is a promising approach to improve transitions from hospital for older adults. Measures of integrated care tend to be survey-based or outcomes focused. This study determined the feasibility of using hospital chart data to measure integrated processes of care.

**Methods:**

This paper reports on two objectives: 1) the development of an integrated care transition framework and associated features of care; 2) a pilot study to test if the features could be applied to 214 hospital patient charts.

**Results:**

Twenty-four features were tested, and fifteen features could be reliably measured using chart review. Of these, the percent of patients classified as receiving integrated care varied widely across the items, from 0.05% to 84.1%.

**Discussion:**

The framework presented in this paper can guide measurement of system and clinical delivery of integrated care transitions. In combination with other tools, chart review can provide perspective on day-to-day care delivery not otherwise accessible, and highlight areas requiring practice change.

**Conclusion:**

Multiple measurement perspectives are needed to improve our understanding of how integrated care is being implemented. While chart review cannot address the full breadth of integrated care, it can help understand how processes of care are being implemented in routine daily care.

## Introduction

The transition from hospital to home is associated with higher rates of adverse events and hospital readmissions for older adults [1,2]. This has resulted in a focus on improving discharge planning interventions for individuals aged 65 and over [3]. Discharge planning is defined as “the development of an individualized discharge plan for a patient prior to them leaving hospital for home”(p. 6) [3] and consists of pre-discharge hospital activities with or without post-discharge support typically provided by hospital affiliated staff [3,4]. Discharge planning interventions initially demonstrated success at reducing hospital lengths of stay and hospital readmissions [3,5]. However, recent studies of discharge planning have shown less improvement in these health service outcomes than has been achieved in the past [6], and intervention success has varied across different institutional and population contexts [7,8,9]. Not only do there continue to be issues with health service outcomes, there also continues to be issues with patient-oriented outcomes. For instance, there are concerns that there is a lack of patient involvement in discharge planning processes [10], and Health Quality Ontario found that 50% Ontarians lacked basic self-care knowledge post-discharge [11].

The plateau of improvement in outcomes when older adults transition from hospital to home suggests that a new approach is needed to address this persistent health care challenge. *Integrated care* is a very promising approach to health and social care that is gaining recognition for providing a higher quality of care for older adults and/or people with chronic disease while maximizing health resource efficiency [12,13]. Integrated care is defined as the application of multiple methods to improve alignment and collaboration between different components of the health and social care sectors to reduce fragmentation, particularly for patients with complex, long-term care needs. Integrated care is a broad concept and includes initiatives that could occur at upper level administrative levels, at the organizational level, or at the direct clinical care level [14]. It weighs equally the system and the patient perspective, thus being a helpful model for discharge planning, where a balance needs to be achieved between pressure on the hospital due to slow or delayed discharges, and the needs of the patient that need to be met to avoid both negative personal (e.g. safety issues) and system (e.g. readmissions) outcomes. Measuring the extent of integrated care occurring in hospital to home transitions may provide insight into how to modify care to improve outcomes.

While there has been a proliferation of measures of integrated care in the last decade [15,16], and it has been argued that a focus of future research should be the validation of existing measures [15], gaps persist. One of these gaps is “whether, and the extent to which integration occurs in the actual delivery of care” (p. 377)[15]. This gap is particularly important because without an understanding of the extent of integration in day-to-day delivery of care, it is not possible to determine the impact of integration on patient and system outcomes [17]. Further, measures developed to date are primarily surveys that rely on patient, provider and administrator recall [15,18]. Finally, while measures that specifically address the integration between hospital and home include components of integrated care, such as care coordination and patient-centredness [18,19], there is little description of the integrated care foundation upon which the measures were developed.

Patient chart review is the most feasible method for examining patient care processes [20]. Patient charts are a readily accessible rich data source that provides more information than can be feasibly collected with primary data collection, and guards against attrition when studying a population experiencing illness [21]. Further, patient charts provide more in-depth information on specific components of day-to-day care than administrative health care data, such as hospital discharge abstracts [21].

This study aimed to determine the feasibility of using hospital chart data to measure integrated processes of care. Two objectives were addressed: 1) develop an integrated care transition conceptual framework and a list of associated features of integrated care for care transitions and, 2) conduct a pilot study to determine if the list of features could be used to measure day-to-day care delivery using chart review.

## Development of a Conceptual Framework of Integrated Care Transitions

Stelfox and Strauss suggest that a conceptual framework is helpful for guiding the development of measures of care processes [22].

### Methods

A critical review approach [23] was used to develop a conceptual framework of integrated care transitions and associated list of features of integrated care transitions. A critical review takes stock of current literature and evaluates what is of value. The reviewer applies a critical eye to diverse sources, and manifests the results into a hypothesis or model [23]. The strengths and weaknesses of literature related to our objective was weighed, and then literature relevant to the objective was integrated into a conceptual framework that was used to derive a list of features of integrated care transitions.

#### Data Collection

An extensive scope of literature was reviewed on the topics of: 1) best practices for hospital discharge planning/care transitions, and 2) integrated care frameworks. The topic of *care transitions* from hospital to home for older adults was searched in PsychInfo and PubMed using the keywords Health AND Transition OR “Discharge Plan*” OR “care transition” and filtering by language (English), age (older adults [65 years and older]), year (2000–2018). Articles were also gathered from the author’s personal literature collections, database alert updates and reference lists of relevant articles. *Integrated care frameworks* were located by broadly searching personal libraries, library databases (PsychInfo and PubMed), the *International Journal of Integrated Care* and a previously published scoping review (reference anonymized for review) of literature on integrated care initiatives for transitions from hospital to community care for older adults. Literature was chosen for further review based on: a) relevance, and b) empirical quality.

#### Development of Framework and Features

The first author immersed themself in both fields of literature simultaneously. Integrated care frameworks were compared and contrasted to explore the similarities and differences. The integrated care frameworks and care transitions literature was also compared and contrasted to explore commonalities and best evidence on care transitions.

The integrated care literature had two levels of frameworks: 1) high-level conceptual frameworks that addressed either a specific population of interest (e.g. people with ongoing care needs [23]), or integration within one setting but not both (e.g. primary care integration [24]) and, 2) intervention models in which the foundational framework was not discussed.

To develop the framework, the foundational concepts of integrated care that are relevant to care transitions for older adults from hospital to home from three sentinel frameworks were identified. The first is the Hollander and Prince *Enhanced Continuing Care Framework (ECCF)* [24], chosen because it focuses on older adults with continuing care needs, our primary population of interest. This framework outlines philosophical and policy prerequisites that provide a base for the development and application of best practices of continuing care. Although it is focused on community-based care, the framework acknowledges linkages with the hospital to be an important component for the delivery of integrated care to this population.

The second framework that informed the framework development was the Kodner and Spreeuwenberg [14] model. It was chosen because of its emphasis on the importance of within-hospital as well as between-hospital integration, given that hospitals tend to decentralize and divide service delivery to manage hospital service complexity [14]. Finally, the Rainbow framework [25] is a very well recognized model of integrated care for primary care. It was included because it emphasizes holistic and interprofessional care, two evidence-based components of high quality care transitions [6,26].

To determine the conceptual foundations in common in these frameworks, the first author mapped the essential components of these frameworks in a matrix, and looked for common conceptual elements across the three frameworks. The following four foundational concepts were identified: a biopsychosocial approach; horizontal integration; vertical integration; and patient-centred care. See ***Table 1*** for an overview of the conceptual foundations of integrated care frameworks and examples of how each conceptual foundation is expressed in each framework.

**Table 1:** Common Conceptual Foundations Across Integrated Care Frameworks and Examples.


CONCEPTUAL FOUNDATION	HOLLANDER AND PRINCE ENHANCED CONTINUING CARE FRAMEWORK (ECCF) [24]	KODNER AND SPREEUWENBERG [14]	RAINBOW FRAMEWORK [25]

***Biopsychosocial approach***	Principle: “A commitment to the psychosocial model of care” (p. 46). Physical and mental health service integration is needed.	“Integration of knowledge and working methods in general medical practice is necessitated by the bio-psychosocial nature of illness.” (p. 2)	A core value is the “integration of the biomedical, psychological and social dimensions of health and well-being.” (p. 8)

***Horizontal integration* (integrating health and social systems of care)**	Social and health services need to be coordinated and linked.	Integrated care relates to the provision of health care, social services and related supports.	“Vertical- and horizontal integration through inter-sectorial partnerships across the health and social service system is needed.” (p. 9)

***Vertical integration* (integrated health systems from primary to tertiary)**	Levels of health care, from primary through to tertiary/quaternary need to be coordinated and linked.	Integration occurs within and between the cure and care sectors.	“Both vertical and horizontal integration are needed to counteract the fragmentation of services in a health system” (p. 4)

***Patient-centredness***	A clinical best practice is the involvement of clients and families. In particular, ensuring clear information provision and communication.	Integrated care is patient-centric: “characteristics and needs of specific patient groups and their ‘fit’ (or lack thereof) with existing systems of care and cure more or less determine the what, how, and where of integration.” (p. 5)	An important feature of integrated care is person-focused care, which is based on “personal preferences, needs, and values which is in contrast to a disease-focused view.” (p. 4)


Next, specific, measurable clinical features of care transitions from three published integrated care lists and taxonomies were identified [26,27,28]. The first source was a scoping review in which clinical elements of integrated care from integrated care for older adults were reported [27]. The second source was a list of clinical features of integrated primary care from an integrated primary care taxonomy developed from a literature review and a Delphi process with health care providers and administrators [28]. The third source was elements from the *Development Model for Integrated Care* developed by Minkman and colleagues using a Delphi process that included 31 experts in integrated care, including researchers, project managers and managers. This source was particularly helpful for operationalization of patient-centred communication features [29].

Two best practice models for care transitions also informed framework development: 1) The Coleman Transitional Model [30], and 2) the Reengineered Hospital Discharge Program [31]. These best practice models ensured the features followed best practice for discharge from hospital.

The features of integrated care and care transitions from these sources were reviewed for relevance to the objective and simultaneously grouped into domains. The domains represent conceptually important elements of integrated care as applied to the context of hospital to home care transitions. Developing these domains ensured that the features that were selected were relevant and inclusive to the context of interest. For example, the literature on care transitions emphasizes the importance of in-hospital integration. Considering how in-hospital integration relates to the foundations of integrated care ensured that these processes were considered in the features checklist. The resulting domains were:

1. *Coordinating care between hospital and community*: This domain is consistent with the foundational integrated care concept of vertical integration across the health care system [13]. In this case, it is about communication, or shared responsibility in care provision with the purpose of providing continuity of care and a high quality plan of care to support care transitions in and out of the hospital.
2. *Individualized multidisciplinary care plan*: This domain is consistent with the foundational integrated care concept of a biopsychosocial approach in integrated care frameworks [24], since care transition evidence indicates that multidisciplinary and individualized care is best practice for care transitions [29]. This domain includes features that allow for the development and implementation of a multidisciplinary discharge plan at the individual client level for care transition needs.
3. *Patient and family involvement in the disposition plan and process*: This domain is consistent with the foundational integrated care concept of patient-centred care. For care transitions, best practice is for clients and families to be pro-actively involved in transition plans [29,30]. Client/family education should be holistic in nature (medical, psychological and social aspects of health). Information on the disposition plan or discharge instructions is unambiguous and understandable at the individual level [28].
4. *Within-hospital coordination for disposition planning*: This domain is conceptually consistent with the concept of horizontal iteration, but is specific to the setting of the hospital to stay true to the setting of interest [14]. This domain speaks to the need for disposition planning to be considered and well-coordinated throughout the stay in order to support timely and safe discharge [29,30].

**Figure 1:**
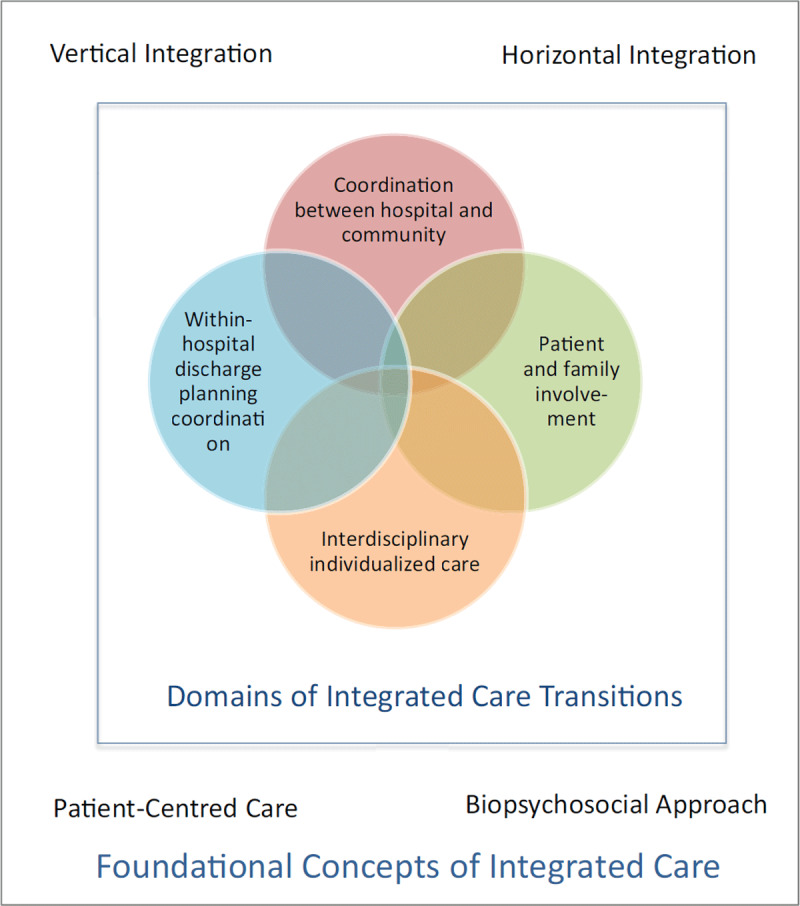
Conceptual Framework for Integrated Care for Care Transitions.

The conceptual framework that includes foundational aspects and specific domains of integrated care transitions is shown in ***Figure 1***.

***Table 2*** shows the four domains of integrated care transitions and the associated features. While some of these features could fit into multiple domains, they have been categorized into the domain with which they fit best for simplicity.

**Table 2:** Domains and Features of Integrated Care Transitions.


DOMAIN OF INTEGRATED CARE TRANSITION CARE	FEATURES OF INTEGRATED CARE TRANSITIONS FROM HOSPITAL TO HOME

**Coordinating care between hospital and community**	When someone is admitted to hospital, their chronic care delivery is conducted by a multidisciplinary team following a care pathway or guideline [27,28,32].

	When someone is admitted to hospital, their care information is transferred to the hospital using a standardized procedure [27,31].

	On admission to hospital, information is shared on the person’s health and social care between the community and the hospital [27].

	The person receives care from a care coordinator that can provide care across settings [27,28,31,32].

	The person receives care from a case manager that provides care in any setting [27,28,31,32].

	The primary care physician is involved in the care during the person’s hospitalization [27,28].

	While the person is in hospital, there is regular communication between community agencies involved in the persons’ care and the hospital [27].

	When someone is discharged to community, their written discharge care plan is transferred from hospital to community using a standardized procedure [27,31].

	Prior to, or within 48 hours of hospital discharge, the person’s individualized care plan is communicated to community providers [27,32].

	The discharge information for community providers includes the social situation and plan to support community care provision [32].

	Cross-boundary person-specific education or training between health care providers is provided [27].

	Follow-up appointments with primary care provider and others are in place at time of discharge [27,30].

	Post-hospital support is provided within 48 hours of discharge to ensure needs being met and determine new needs [27,32].

**Interdisciplinary individualized care**	The person receives care from a multidisciplinary team providing care across settings [27].

	The person received multi-domain assessment of discharge needs and a plan to meet these needs in hospital [27,28,32].

	Hospital disposition planning by a multidisciplinary team follows a care pathway or guideline [27,28,32].

**Within-hospital coordination**	The person’s risk is assessed to determine the level of care transition support needed during hospitalization [27,32].

	The client has provider continuity during the hospital stay, by means of an assigned care coordinator [27,31].

	The person’s health and social care needs for discharge is discussed at regular multidisciplinary meetings [27].

**Patient involvement in care and care planning**	The person and their family are involved in the discharge planning process [27,30,31].

	The person and their family’s preferences are incorporated into the discharge plan to ensure their satisfaction [28,30].

	Client and family provided with education about reason for medical stay and self-care instructions to follow on discharge [27,28].

	The discharge instructions are individualized to the person’s knowledge needs to ensure understanding [19,25].

	Client is referred to a post-discharge self-management program [28,32].

	Client’s discharge care needs are met regardless of program eligibility requirements [32].


## Pilot Study

The purpose of the pilot was to determine if the list of features of integrated care could be used to measures processes of integrated care in day-to-day clinical practice.

### Methods

The method was chart review – a common way to assess quality in routine patient care [33]. Patient chart review is a way to access rich data that provides more information than can be feasibly collected with primary data collection [20,21]. Further, patient charts provide more in-depth information than administrative health care data, such as hospital discharge abstracts [21]. This study was approved by the local Health Research Ethics Board.

#### Setting and Sample

The sample was taken from a teaching hospital in a Canadian province. Canada has a universal health care system in which physician and hospital services are provided free of charge. The health region of this study has some elements of integrated care at the macro level, such as overarching governance, and an administrative structure that operates across acute care centres. However, it has received critique for focusing too much on acute care to the detriment of the continuum of care. One reason for this imbalance may be because there is no integration of governance between the “cure and care sectors” (p. 3) [14], thus this system continues to struggle with coordination and continuity between tertiary and primary care [34]. The study hospital is located in a core urban area, and serves a dual role of being a teaching and community hospital.

Data were collected from 214 hospital charts of older adults who were under the care of the general medicine service of the study hospital. Inclusion criteria were: age 65 or over at the time of hospital admission, and living at home preadmission. To focus on the concept of integrated care, patients who would most benefit from an integrated approach were targeted, i.e. those requiring continuing care after discharge. This included those discharged home with home care (71.0%), or transferred to institutional care (inpatient rehabilitation or nursing home). Patients who died during the hospitalization were excluded. Starting in December 2016, charts that met the study inclusion and exclusion criteria were pulled backwards in time, until the desired sample (minimum of 200) was reached. This cohort approach provided a snapshot of clinical practice in a specific time period. All patients had a discharge date between January 2014 and September 2016.

#### Data collection

*Personal characteristics*. For descriptive purposes, information was collected on patients’ personal characteristics including demographic information (age, sex, income, language), social information (whether or not the patient lived alone and the presence of an informal care provider, preadmission home care enrolment) and function (cognitive impairment and mobility assistance required preadmission and upon hospital discharge). More detailed information on the definitions of these variables and the data collection method are provided elsewhere [36].

*Integrated care transition*. Data in ten patient charts were reviewed first to consider if and how the chart data could be adapted into measureable items. Six of the 24 features could not be used because:1) the feature was not present in the health care setting (e.g. there were no standardized processes to share community-based information with the hospital upon admission); or, 2) the feature could not be measured using chart data (e.g. family and patient satisfaction). Three features were adapted slightly to the setting so that they could be measured. For example, for the feature “post-hospital support is provided within 48 hours of discharge to ensure needs being met and determine new needs”, the number of people who had their home care services in place at the time of discharge were counted.

For the remaining 18 features, quantitative as well as qualitative data was collected from the 214 charts to derive measurable items. Extraction procedures for the chart review used multiple strategies to ensure data fidelity and were informed by guidelines developed by Gearing and colleagues [21], and Allison and colleagues [35], the details of which have been described elsewhere [36]. See Appendix for detail on the data source in the chart for each feature.

#### Data Analysis

The proportion of missing values, and the proportion of patients for whom each integrated care item had been met was calculated for all 214 charts. The inter-rater reliability (IRR) using thirty charts (14% of the sample) was calculated using Cohen’s kappa as well as the percent agreement to aid in interpretation [37]. A Cohen’s kappa of >.60 is considered acceptable in the literature [37]. For percent agreement, there is consensus that 95% agreement is high quality [35,37], but there is little guidance on lower levels of acceptability. Therefore, 80% agreement and over was considered acceptable since this was a pilot study.

### Results

*Personal characteristics*. Approximately half the sample was over 80 years old (47.2%), lived in a low-income neighbourhood (49.5%), and lived alone (49.5%). As is typical with an older adult population, women were over-represented, with 59.3% of the sample being female. Approximately three quarters of the sample spoke English at home (76.6%), and had at least one identified an informal care provider (74.3%). Most of the patients were already known to community health services, with 90.2% enrolled in the publicly funded home care program. Approximately a third had documented cognitive impairment both preadmission (35.0%) and at discharge (30.8%). Fifteen percent of the sample required assistance to mobilize at hospital admission, and 37.9% needed help to mobilize upon hospital discharge.

*Measure of Integrated Care*. After excluding one item that did not meet our inter-reliability standard of >80%, we had remaining a set of 17 reliable items. Of these 17 items, percent of patients who were classified as having integrated care varied widely across the items, from 0.05% to 84.1% (see ***Table 3***).

**Table 3:** Results of integrated care items from chart review.


ITEM	% PATIENTS WHO MET ITEM

*Coordinating Care between Hospital and Community*	

1. There is a community care coordinator actively involved in the client’s care as measured by recent community assessment available.	57.8

2. The client has a primary care physician (PCP) that is recorded upon hospital admission.	70.6

3. While the person is in hospital, there is communication between community agencies involved in the persons’ care and the hospital (excluding home care).	27.1

4. Discharge summary was cc’ed to the PCP or receiving institution.	56.6

5a. Prior to, or within 48 hours of hospital discharge, the person’s discharge summary is available for receiving institution/care provider.	29.0

5b. Prior to, or within 48 hours of hospital discharge, the person’s discharge prescription is faxed directly to pharmacy.	70.1

6. Follow-up appointments with primary care provider are in place at time of discharge for those going home.	32.2

7. Cross-boundary person-specific education or training between hospital and community health care providers is provided for discharge care.	0.05

8. All post-hospital recommended home care in place upon hospital discharge.	76.2

*Interdisciplinary Individualized Care*	

9. Preadmission, the client received care from a community-based or boundary-crossing multidisciplinary team.	22.0

10. The person’s risk is assessed to determine the level of care transition support needed during hospitalization (using hospital discharge screening tool).	77.5

11. The client receives a multi-domain assessment of discharge needs in hospital (multidisciplinary team working with client includes both social and health care professionals).	80.8

*Within-Hospital Coordination*	

12. Disposition planning of multidisciplinary follows a care pathway or guideline (as developed with discharge risk tool).	36.0

13. The person’s health and social care needs for discharge is discussed at regular multidisciplinary meetings.	76.6

*Patient Involvement in Care Planning*	

14. The discharge plan is discussed with the family.	69.7

15. Client provided with written discharge information form.	81.6

16. The discharge instructions are free of jargon.	46.1


## Discussion

The purpose of this study was to determine the feasibility of using hospital chart data to measure integrated processes of care. First an integrated care framework and associated features of integrated care was developed using literature in the fields of integrated care and care transitions. Then it was determined if these features could be used to extract information about integrated care delivery from hospital patient charts.

The integrated care features developed here could be used in two different ways by organizations. First, organizations can use the features as a checklist to consider the extent of clinical integration of care at a systems level and determine areas for development. For example, this pilot revealed gaps in care delivery as there were some elements of care not present in this health system, and therefore could not be measured at an individual level. For example, the use of specific procedures for sharing information from the community to the hospital on admission, or the use of system-wide pathways or protocols. The second way that organizations can use these features is for a quality improvement chart review For example, in the setting in this study, the chart review showed gaps between best practice and standard daily care delivery for communication at discharge [38,39]. Only 46.1% of the discharge instruction sheets distributed to patients had the instructions written without hospital jargon (e.g. DAT for diet as tolerated), and the discharge summary was available for sharing with other health providers within 48 hours of hospital discharge only 29% of the time.

This study illustrates that chart review alone is not adequate to understand the full extent of integrated care practices being delivered. For example, measuring concepts of relational continuity, the individualization of care, and whether or not care was patient-centred in a reliable fashion were not possible with chart review. These concepts require an understanding of the patient perspective of care that cannot be gleaned from a chart review. Thus, the use of a patient-oriented measure is also needed in determining the extent that patient’s integrated care needs are being met, such as the Coleman Transition Measure [30].

For these chart review items to be used in future research they require validity testing [15]. The validity testing needs to address both patient and system perspectives to align with the two-pronged goals of integrated care [14]. Patients, families and health system administrators need to be consulted to determine the face validity of these measures. Further, construct validity would need to be established by correlating these measures to patient and system outcomes such as patient satisfaction and hospital readmissions. Further, since chart review methodology was found to address only some components of robust integrated care models, further testing would be required to see how this set of measures could complement other already existing measures. For example, the use of these measures in tandem with a patient-oriented measure such as the Coleman Transitions Measure [19] may provide a well-rounded understanding of the application of integrated care principles in the hospital setting that either tool cannot provide alone.

Another area for future methodological research is the use of narrative chart data. There was narrative chart data available that provided an understanding of the quality of care being delivered but that we were unable to extract and measure reliably. Developing sound methods for this type of data extraction is an area for future work, as chart review method literature focuses primarily on collection of discrete quantitative variables [21], rather than narrative data that is more qualitative in nature. Such narrative data could provide valuable insights into quality of care delivery.

This study has several limitations. To address objective 1, while the review of relevant literature was extensive and systematic for some components, not all components of the search were systematic. Experts were not consulted on the conceptual framework developed in this study, although it drew on high quality empirical literature that included expert consultation. For objective 2, although this study focused on care transitions between the hospital and the home, and considering the community and hospital processes simultaneously is important, we only used hospital chart data. It was not feasible to include primary care data, as multiple primary care sites served the patients in this study site. The data may have been more robust if data had been included from primary care practitioners, and future validation work should address this possibility in order to determine if needs are met post-discharge. However, this hospital snapshot can help with gaining perspective on the hospital processes that are occurring and how they fit into the bigger health system context in relation to integrated care. Further, as discussed, this study did not include validity testing of the features list. Despite these limitations, this study provides information that may be helpful to researchers and policy-makers who wish to measure individual level integrated care practices in their health care settings.

## Conclusion

Integration is key to improving the quality of care transitions for older adults. The list of integrated care features developed here can support systems in determining ways to improve the extent of integration occurring at the clinical system and individual level, in conjunction with other patient oriented care transition tools. While chart review cannot address the breadth of integrated care, it can help understand how processes of care are being implemented in routine daily care.

## Additional File

The additional file for this article can be found as follows:

10.5334/ijic.5552.s1 Appendix. Data Sources [Data source from chart for each of the features of integrated care piloted].
