# Supplementary material for: Measuring Processes of Integrated Care for Hospital to Home Transitions
Source: Int J Integr Care. 2021 Apr 26;21(2):12. doi: 10.5334/ijic.5552 (PMC8086722; doi:10.5334/ijic.5552)
Supplement: Appendix. — Data Sources [Data source from chart for each of the features of integrated care piloted]. [file ijic-21-2-5552-s1.pdf]

Appendix: Data sources

Features of Integrated Care Transitions from Hospital to Home (As listed in Table 2 Manuscript)	Data source in chart to measure?	Wording of measure tested	Inter-Rater Reliability % agreement/kappa
When someone is admitted to hospital, their chronic care delivery is conducted by a multidisciplinary team following a care pathway or guideline [17, 22, 26].	No, this element is not present in the health care setting. Would not be any variation between individuals.	N/A	N/A
When someone is admitted to hospital, their care information is transferred to the hospital using a standardized procedure [17, 25].	No, this element is not present in the health care setting. Would not be any variation between individuals.	N/A	N/A
On admission to hospital, information is shared on the person's health and social care between the community and the hospital [17].	Data was not in consistent format.	N/A	N/A
The person receives care from a care coordinator that can provide care across settings [17, 22, 25, 26].	For those with home care pre-admission, *Home Care Community Report* placed on chart to provide preadmission status.	**1. There is a community care coordinator actively involved in the client's care as indicated by there being a recent community assessment available (within last 6 months prior to hospital admission).**	96.7/.93
The person receives care from a case manager that provides care in any setting [17, 22, 25, 26].	*Home Care Community Report*; *In-Hospital Discharge Screening Form.*	The person receives care from a private or public agency that has provided a case manager	86.7/.44
The primary care physician is involved in the care during the person's	*Hospital Admission Form*: "Name of PCP"	**2. The client has a primary care physician (PCP) that is**	100/1.00

hospitalization [17, 22].		**recorded upon hospital admission.**	
While the person is in hospital, there is regular communication between community agencies involved in the persons' care and the hospital [17]	Interprofessional Progress Notes; *Weekly Discharge Rounds* form	**3. While the person is in hospital, there is communication between community agencies involved in the persons' care and the hospital (excluding home care).**	83.3/.86
When someone is discharged to community, their written discharge care plan is transferred from hospital to community using a standardized procedure [17, 25].	*Discharge Summary*	**4. Discharge summary was cc'ed to the PCP or receiving institution.**	83.3/.66
Prior to, or within 48 hours of hospital discharge, the person's individualized care plan is communicated to community providers [17, 26].	a) *Discharge Summary* (date of transcription)<br>b) Nursing discharge note in interprofessional progress notes; Fax Sheet to Pharmacy; *Discharge Information Form*	**5. a) Prior to, or within 48 hours of hospital discharge, the person's discharge summary is available for the receiving institution/care provider.**<br>**b) Prior to, or within 48 hours of hospital discharge, the person's discharge prescription is faxed directly to pharmacy.**	a) 96.7/.93<br>b) 93.3/.90
The discharge information for community providers includes the social situation and plan to support community care provision [26]	*Discharge Summary*	Discharge summary provides social situation and plan to support community care.	66.7/.33
Follow-up appointments with primary care provider and others are in place at	Only able to consistently track PCP appts.	**6. Follow-up appointments with primary care provider**	90.0/.80

time of discharge [17, 24]	Discharge Nursing Note; *Patient Information Discharge Form*	**are in place at time of discharge for those going home.**	
Cross-boundary person-specific education or training between health care providers is provided [17].	Interprofessional progress notes	**7. Cross-boundary person-specific education or training between hospital and community health care providers is provided for discharge care.**	Unable to calculate due to only one case with this criterion.
Post-hospital support is provided within 48 hours of discharge to ensure needs being met and determine new needs[17, 26].	Interprofessional progress notes. Measure accommodates data collection method.	**8. All post-hospital recommended home care is in place upon hospital discharge.**	93.3/.93
The person receives care from a multidisciplinary team providing care across settings [17]	*Home Care Community Report*; Admission Notes; Occupational or Physical Therapy discharge assessment notes. Measure adapted to account for the fact that there are no multidisciplinary teams in this system that provide care across settings.	**9. Preadmission, the client received care from a community-based or boundary-crossing multidisciplinary team.**	93.3/.83
The person's risk is assessed to determine the level of care transition support needed during hospitalization [17, 26].	*Discharge Screening Form* that categorizes patients into high, medium and low risk for readmission to help identify patient needing more discharge planning support.	**10. The person's risk is assessed to determine the level of care transition support needed during hospitalization (using hospital discharge screening tool).**	100/1.00
The person received multi-domain assessment of discharge needs and a plan to meet these needs in hospital [17,]	*Interprofessional Consultation Form* and/or Interprofessional progress notes. Being involved in care was	**11. The client receives a multi-domain assessment of discharge needs in**	96.7/.87

[22, 26].	defined as: provided at least consultation to the team (indirect) or provided at least one direct visit.	**hospital (multidisciplinary team working with client includes both social and health care professionals).**	
Hospital disposition planning by a multidisciplinary team follows a care pathway or guideline [17, 22, 26].	Section on Discharge *Screening Form* (professionals needed for discharge planning). We correlated this plan with the professionals actually providing care during hospitalization using *Interprofessional Consultation Forms* and/or Interprofessional progress notes.	**12. Disposition planning of multidisciplinary team follows the care guideline (on the discharge screening form).**	83.3/.70
The client has provider continuity during the hospital stay, by means of an assigned care coordinator [17, 25].	Feature not present in setting	N/A	N/A
The person's health and social care needs for discharge is discussed at regular multidisciplinary meetings [17].	*Weekly Discharge Planning* form. Recorded presence of form and dates of entries.	**13. The person's health and social care needs for discharge is discussed at regular multidisciplinary meetings.**	100/1.00
The person and their family are involved in the discharge planning process [17, 24, 25].	Interprofessional progress notes and/or narrative notes on *Weekly Discharge Planning* form. Unable to determine patient involvement so measure is for family.	**14. The discharge plan is discussed with the family.**	90.0/.67
The person and their family's preferences are incorporated into the discharge plan to ensure their satisfaction [22, 24].	Unable to measure.	N/A	N/A
Client and family provided with	Patient Discharge Information form.	**15. Client provided with**	93.3/.88

education about reason for medical stay and self-care instructions to follow on discharge [17, 22].	Unable to determine content or quality of the education provided so measure is presence of form.	**written discharge information form.**	
The discharge instructions are individualized to the person's knowledge needs to ensure understanding. (Coleman, Valentijn et al., 2015)	*Patient Discharge Information* form. Jargon examples: DAT, AAT, f/u.	**16. The discharge instructions are free from jargon.**	86.7/.80
Client is referred to a post-discharge self-management program. [22, 26].	Not measureable.	N/A	N/A
Client's discharge care needs are met regardless of program eligibility requirements [26].	Not measureable.	N/A	N/A

Table 1: Domains of Domains and Features of Integrated Care Transitions

Features of Integrated Care Transitions from Hospital to Home from the Literature	Data source in chart to measure?	Wording of measure tested	Inter-Rater Reliability % agreement/kappa
**Measurable and Adequate Inter-rater Reliability**			
1. The person receives care from a care coordinator that can provide care across settings (Brown & Menec, 2018a; Minkman et al., 2009; Valentijn et al., 2015; Jack et al. 2009).			
2 The primary care physician is involved in the care during the person's hospitalization (Brown & Menec, 2018a; Valentijn et al., 2015).			
3. While the person is in hospital, there is regular communication between community agencies involved in the persons' care and the hospital (Brown & Menec, 2018a)			
4. When someone is discharged to community, their written discharge care plan is transferred from hospital to community using a standardized procedure (Brown & Menec; Jack et al., 2009)			
5. Prior to, or within 48 hours of hospital discharge, the person's individualized care plan is communicated to community providers (Brown & Menec, 2018a; Minkman et al., 2009).			
6. Follow-up appointments with primary care provider and others are in place at time of discharge (Brown & Menec, 2018a; Coleman, Parry)			

Features of Integrated Care Transitions from Hospital to Home from the Literature	Data source in chart to measure?	Wording of measure tested	Inter-Rater Reliability % agreement/kappa
7. Cross-boundary person-specific education or training between health care providers is provided (Brown & Menec, 2018a).			
8. Post-hospital support is provided within 48 hours of discharge to ensure needs being met and determine new needs (Brown & Menec, 2018a; Minkman et al., 2009).			
9. The person receives care from a multidisciplinary team providing care across settings (Brown & Menec, 2018)			
10. The person's risk is assessed to determine the level of care transition support needed during hospitalization (Brown & Menec, 2018a; Minkman et al., 2009)			
11. The person received multi-domain assessment of discharge needs and a plan to meet these needs in hospital (Brown & Menec, 2018a; Minkman et al., 2009; Valentjin et al., 2015).			
12. Hospital disposition planning by a multidisciplinary team follows a care pathway or guideline (Brown & Menec, 2018a, Minkman et al., 2009, Valentjin et al., 2015)			
13. The person's health and social care needs for discharge is discussed at regular multidisciplinary meetings (Brown & Menec, 2018a).			

Features of Integrated Care Transitions from Hospital to Home from the Literature	Data source in chart to measure?	Wording of measure tested	Inter-Rater Reliability % agreement/kappa
14. The person and their family are involved in the discharge planning process (Brown & Menec ; Jack; Coleman)			
15. Client and family provided with education about reason for medical stay and self-care instructions to follow on discharge (Brown & Menec, 2018a; Valentjin et al., 2017).			
16. The discharge instructions are individualized to the person's knowledge needs to ensure understanding. (Coleman, Valentijn et al., 2015)			
**Not Measurable**			
1. When someone is admitted to hospital, their chronic care delivery is conducted by a multidisciplinary team following a care pathway or guideline (Brown & Menec, 2018a; Minkman et al., 2009; Valentjin et al., 2015).			
2. When someone is admitted to hospital, their care information is transferred to the hospital using a standardized procedure (Brown & Menec; Jack et al., 2009).			
3. On admission to hospital, information is shared on the person's health and social care between the community and the hospital (Brown & Menec, 2018a).			
24. Client is referred to a post-discharge self-management program. (Minkman et al.,			

Features of Integrated Care Transitions from Hospital to Home from the Literature	Data source in chart to measure?	Wording of measure tested	Inter-Rater Reliability % agreement/kappa
2009; Valentijn et al., 2015).			
25. Client's discharge care needs are met regardless of program eligibility requirements (Minkman et al., 2009).			
18. The client has provider continuity during the hospital stay, by means of an assigned hospital care coordinator (Brown & Menec, 2018a; Jack).			
21. The person and their family's preferences are incorporated into the discharge plan to ensure their satisfaction (Parry; Valentijn et al., 2015).			
**Inadequate Inter-rater Reliability**			
7. The person receives care from a case manager that provides care in any setting (Brown & Menec, 2018a; Minkman et al., 2009; Valentijn et al., 2015; Jack et al. 2009).			
10. A shared multidisciplinary report is transferred to community providers (Minkman, year).			
